# 1440. Hand Hygiene in Nursing Homes During And After The Covid-19 Pandemic Outbreak

**DOI:** 10.1093/ofid/ofad500.1277

**Published:** 2023-11-27

**Authors:** Ida H Sandbekken, Borghild Løyland, Åsmund Hermansen, Inger Utne, Ellen Karine Grov

**Affiliations:** OsloMet, Oslo, Oslo, Norway; OsloMet, Oslo, Oslo, Norway; OsloMet, Oslo, Oslo, Norway; OsloMet, Oslo, Oslo, Norway; OsloMet, Oslo, Oslo, Norway

## Abstract

**Background:**

Residents in Norwegian nursing homes are vulnerable and fragile, and hand hygiene adherence is too low to prevent transmission of all infections. This study aimed to investigate hand hygiene in nursing homes during and after the COVID-19 pandemic, and if interventions targeting behavior change can improve adherence.

**Methods:**

Observations of hand hygiene were collected in February and March 2021 and September and October 2022, using the WHO observation tool. The observations were carried out by trained nursing students and researchers. The first observations were during the pandemic when it was strict regulations in nursing homes, and the last observations were when there were no regulations related to COVID-19. Over 12 months, from August 2021 to August 2022, three of the 22 participating nursing home wards implemented interventions to improve hand hygiene. Multimodal interventions targeting behavior change, including education, UV-light box, and posters, were implemented.

**Results:**

Hand hygiene adherence in total was 58.3% during the pandemic and decreased to 52.5% after the pandemic. The control wards experienced a decrease from 59.5% in 2021 to 51.3% in 2022, while the intervention wards experienced an increase from 54.7% to 60.9% (p-value: < 0.001). Hand hygiene adherence in total was lower when wearing gloves (32.8% vs. 61.7%), and inappropriate glove use was significantly higher after the pandemic. Hand hygiene adherence was 35.3% when using gloves in 2021, in 2022 the adherence had decreased to 27.7% (p-value: < 0.001).

**Figure:**
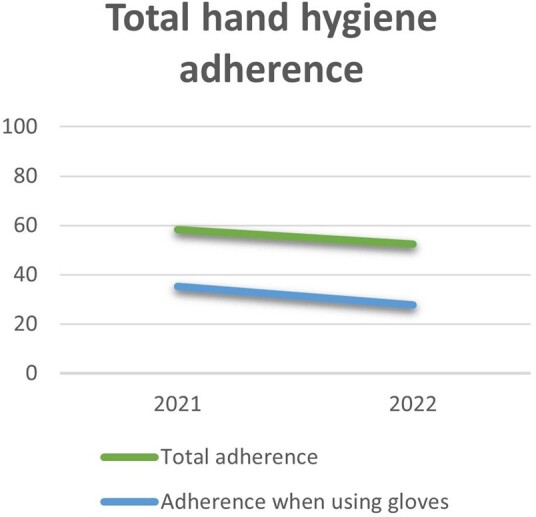

**Figure:**
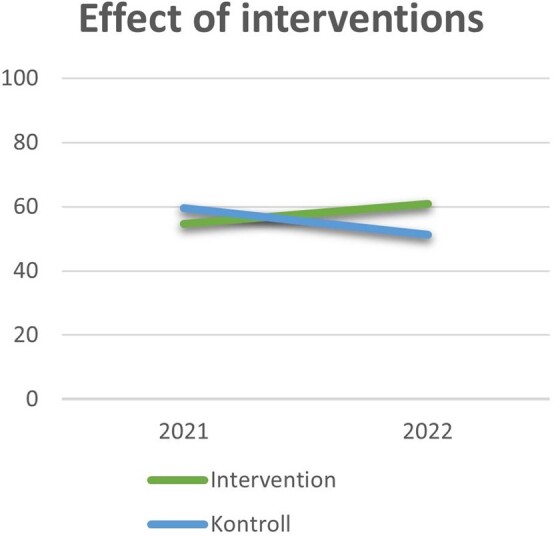

**Conclusion:**

This study shows that the implementation of interventions aimed at behavioral change increased hand hygiene adherence in nursing home wards. Despite hand hygiene adherence being lower, and inappropriate glove use higher than hoped for during the pandemic, we can see that attention and focus on hand hygiene during the pandemic probably had an increased effect on adherence. Attention to hand hygiene and activation of the motivation of healthcare workers is crucial, for receiving an effect of interventions. Hand hygiene adherence decreased after the pandemic when the focus on hand hygiene diminished. Behavior is likely to decline over time, and continuous reinforcement is needed to maintain a behavior change.

**Disclosures:**

**All Authors**: No reported disclosures

